# Cerebral Venous Sinus Thrombosis Manifesting as a Recurrent Spontaneous Subdural Hematoma: A Case Report

**DOI:** 10.1016/j.ijscr.2020.01.059

**Published:** 2020-02-06

**Authors:** Reem Alharshan, Hammad U. Qureshi, Abdullah AlHada, Muhammed Shaikh, Ayman Khalil

**Affiliations:** a King Abdulaziz Hospital, Al Ahsaa, Saudi Arabia; b Department of Neurosurgery, King Abdulaziz Hospital, Al Ahsaa, Saudi Arabia

**Keywords:** CT, computed tomography, CVST, cerebral venous sinus thrombosis, MRI, magnetic resonance imaging, MRV, magnetic resonance venography, SDH, subdural hematoma, Cerebral venous sinus thrombosis, Subdural hematoma, Craniotomy, Anticoagulation

## Abstract

**Background:**

Diagnosing cerebral sinus venous thrombosis (CSVT) manifested as a spontaneous subdural hematoma (SDH) is challenging due to variability of its clinical features. The neuroradiological investigation is crucial to confirm the diagnosis. The management of SDH secondary to CVST is controversial and not well established.

**Case description:**

We present a case of an adult man with Down’s syndrome who underwent a left-sided craniotomy and evacuation of spontaneous subdural hematoma. Post-surgery magnetic resonance venography (MRV) revealed venous sinus thrombosis in the right transverse sinus with loss of flow signal. He was started on Apixaban two weeks post-surgery. Three months later, the patient re-presented with recurrence of the SDH. His anticoagulation was discontinued, and he underwent craniotomy reopening and evacuation of the recurrent hematoma. The patient returned to his baseline following this procedure. The patient was followed up in the out-patient clinic regularly. Interval brain MRI and MRV performed at six months showed further resolution of the thrombosis of the right sigmoid sinus with restoration of the venous flow.

**Conclusion:**

The management of SDH complicating CVST remains controversial due to the rarity of its presentation and the hazards associated with the use of anticoagulation.

## Highlights

• Cerebral sinus venous thrombosis is a rare cause for a subdural hematoma and should be considered in the differential diagnosis of such cases.
• Neuroradiological imaging has a valuable role in diagnosing and follow-up of patients.
• Management of subdural hematoma secondary to venous sinus thrombosis could be challenging due to the controversy in using anticoagulation medications.

## 1 Introduction

Cerebral venous sinus thrombosis (CVST) is a rare, life-threatening condition [1]. The diagnosis of CVST might be underestimated because of significant variability in presentation and the variability of computed tomography (CT) and magnetic resonance imaging (MRI) findings [2]. The management of CVST is challenging, as there are conflicting requirements of anticoagulation for CVST management against the risk of subdural hemorrhage expansion or recollection [3]. Herein, we present a case of CVST diagnosed following a spontaneous subdural hematoma (SDH). This case report has been reported in line with the SCARE criteria [4].

## 2 Case presentation

We report a case of a 23-year-old male, known case of Down’s syndrome, who presented to our Emergency Department with a complaint of severe headache for three days. It was accompanied by nausea, vomiting, and confusion. There was no history of trauma, seizures, loss of consciousness, change in vision, or relevant signs of virus infection. He was not on any regular medications. His medical background included surgical closure of ventricular septal defects in his childhood. There was no past medical or family history of coagulopathies.

**Fig. 1:**
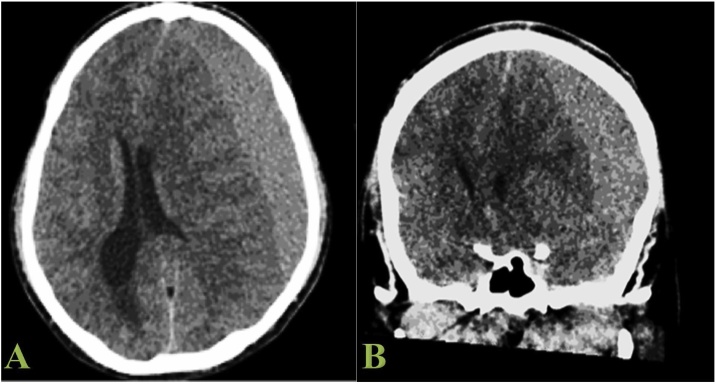
Brain CT coronal (1A) and axial (1B) views showed left sub-acute SDH.

On examination, his Glasgow coma score was 14, with normal vital signs. On neurological examination, the patient was confused, with dense right-sided hemiparesis. Liver function test, prothrombin time, partial thromboplastin time, and international normalized ratio were all within normal range. The brain CT scan revealed left-sided subacute SDH with a 12 mm midline shift and uncal herniation (Fig. 1).

**Fig. 2:**
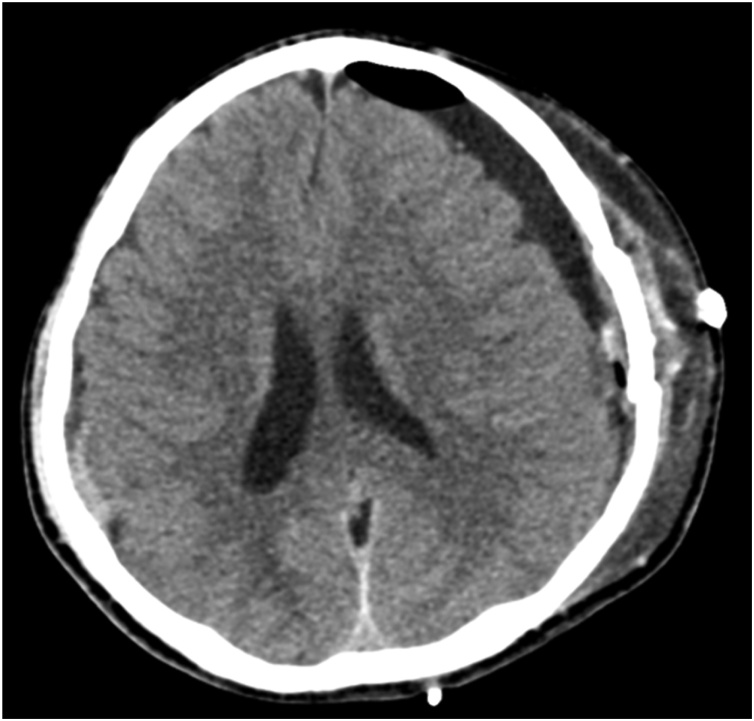
Post-operative CT showing optimum evacuation of left-sided hematoma and a small right-sided SDH.

**Fig. 3:**
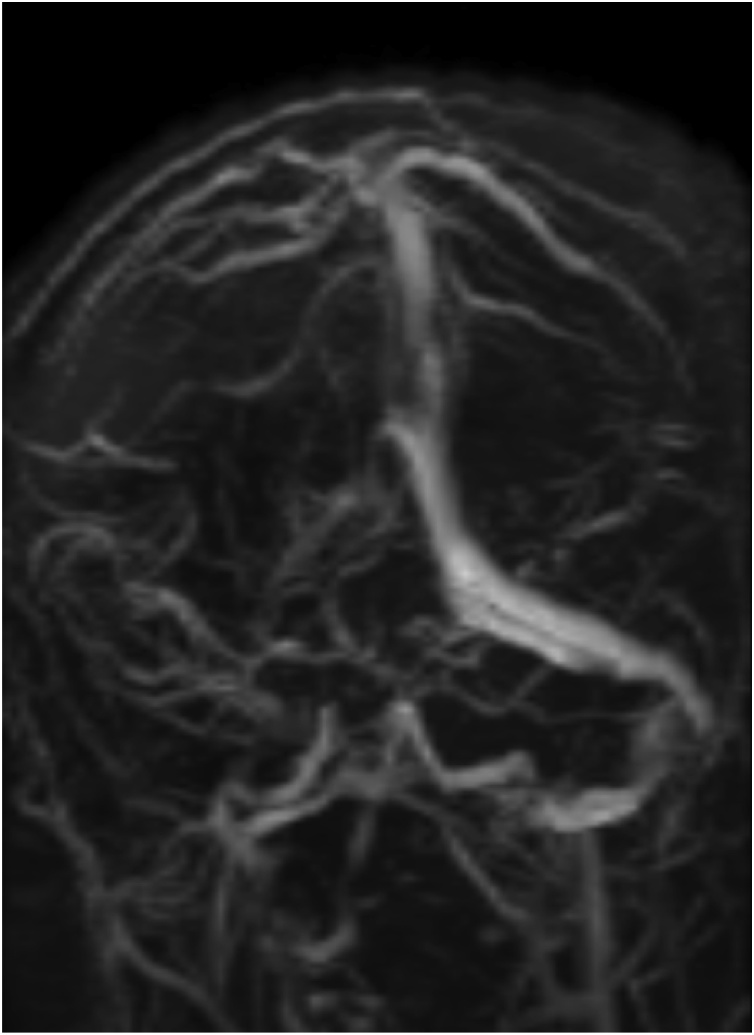
MRV revealed thrombosis in the right transverse and sigmoid sinuses.

The patient underwent emergent left-sided craniotomy and SDH evacuation. The blood evacuated was a mixture of dark stained blood and clots. Post-surgery, the patient’s consciousness level returned to normal, and he regained full power in all limbs. His post-operative CT scan showed optimal evacuation of the left-sided hematoma. There was also a new finding of a small right-sided SDH in the parietal region with no significant mass effect (Fig. 2). The CT scans review showed hyperdense areas in the transverse and sigmoid sinus; this raised suspicion of thrombosis. Magnetic resonance venography (MRV) confirmed the diagnosis of CVST in the right transverse and sigmoid sinuses with loss of flow signal (Fig. 3).

Clinically, the patient made an excellent post-operative recovery. After the diagnosis of CVST, we obtained neurology opinion and considered it risky to commence therapeutic anticoagulation in the early post-operative phase. He was started on a prophylactic dose of low molecular heparin. The patient was discharged home one week after surgery. Subsequently, he was readmitted electively two weeks later and was commenced on Apixaban. He was followed up regularly in outpatients and was asymptomatic.

**Fig. 4:**
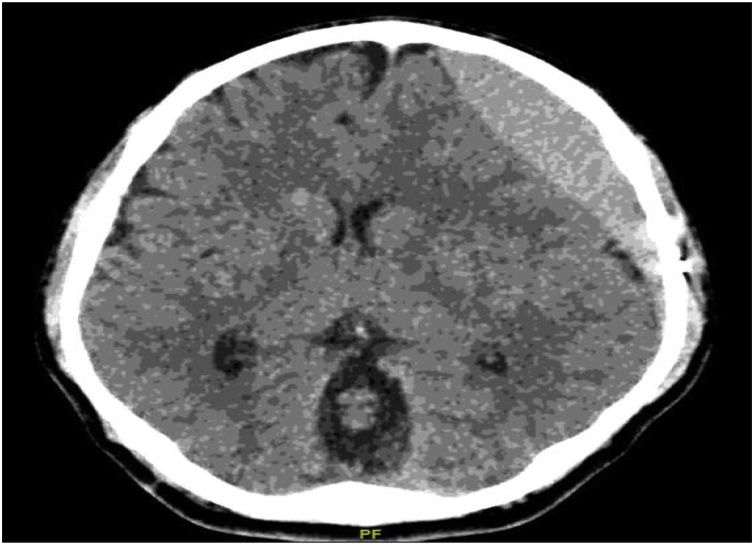
CT brain following the second presentation showing recollection of hematoma in the left subdural space.

The patient re-presented three months later complaining of a headache and recurrent vomiting, with normal consciousness levels. His repeat brain CT showed recollection of hematoma in the left subdural space (Fig. 4). An updated MRV revealed an interval shrinkage of the thrombus in the right sigmoid venous sinus; however, the reduced flow was seen through the sinus. The medical decision was to stop the anticoagulation, and the patient underwent reopening of the left-sided craniotomy and evacuation of the recurrent SDH. He recovered well after surgery, and we elected not to restart anticoagulation and follow-him clinically.

**Fig. 5:**
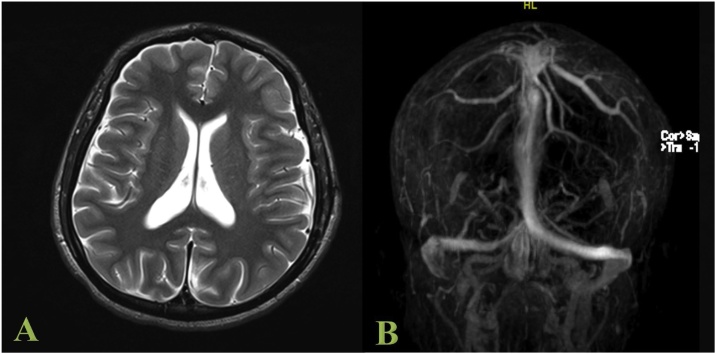
MRI (A) and MRV (B) brain performed six months following the second surgery for evacuation of SDH recollection showed complete resolution of the SDH, with residual partial thrombosis of the right sigmoid sinus; however, the sinus is patent and the venous flow restored.

The patient remains clinically well. Interval MRI and MRV scans at six months show complete resolution of the SDH, with residual partial thrombosis of the right sigmoid sinus; however, the sinus is patent, and the venous flow is restored (Fig. 5).

## 3 Discussion

Despite an extensive literature search, CVST presenting with spontaneous SDH is rare, and the diagnosis may be easily overlooked [5]. In the International Study on Cerebral Vein and Dural Sinus Thrombosis, none of the patients (n = 624) were reported to have SDH [6]. On the other hand, a prospective neurosurgical database identified only three patients diagnosed by CVST manifested with SDH over six years [3].

The mechanism underlying the onset of CVST in Down’s syndrome is unclear [7]. Wilcock et al. identified that in Down’s syndrome, there are three abnormal proteins encoded on chromosome 21 that might trigger superoxide dismutase 1, Interferon-gamma receptor, and cystathionine synthase; These abnormal proteins are responsible for activating pro-inflammatory mediators and complement system, which could lead to potential thrombogenesis [8].

In our case report, we demonstrate a rare case of CVST diagnosed following investigations for a Down’s syndrome patient presented with spontaneous SDH. To date, there are only five published case reports of CVST in patients with Down’s syndrome [7,[9], [10], [11], [12]]. None of these cases had SDH as a manifestation of the CVST.

The first-line management for CVST is systemic anticoagulation [13]. However, the initiation of therapeutic anticoagulation must be carefully weighed against the potential risk of intracranial hemorrhage [14]. The diagnosis of CVST combined with SDH presentation is a challenging case to manage. Khatib et al. suggest an algorithm to treat this complication by starting immediate anticoagulation if the patient is stable and has no mass effect or midline shift in neuroimaging [15]. However, if the patient has a neurological deficit with mass effect or midline shift in the neuroradiological investigation, then immediate neurosurgical intervention with supportive care is recommended [15]. They recommend re-evaluation of the patient for the risk of rebleeding two weeks post-operatively and consider oral anticoagulation [15]. In our case, we elected to apply this algorithm, and we considered a satisfactory outcome for three months. However, after the recurrence of SDH, we considered the risks of restarting the anticoagulation are higher than the expected benefits, so we elected to manage the patient expectantly with clinical and radiological follow-up without anticoagulation medications.

## 4 Conclusion

Spontaneous SDH secondary to CVST is a rare condition; however, it should be included in the differential diagnosis in patients presenting with non-traumatic subdural hematoma. Accurate and prompt management is required to prevent both acute and long-term consequences. The role of anticoagulation treatment in CVST patients complicated by intracranial bleeding remains a controversial issue, and guidelines need to be developed.

## Declaration of Competing Interest

No known conflicts of interest.

## Funding

No sponsors of any kind for the case report.

## Ethical approval

Case reports exempt from ethical approval from the institution.

## Consent

Written informed consent was obtained from the patient for publication of this case report and accompanying images. A copy of the written consent is available for review by the Editor-in-Chief of this journal on request.

## Authors contribution

Reem AlHarshan: Acquisition, analysis, and interpretation of data, drafting the article, final approval of the version to be submitted.

Ayman Khalil: Surgeon, involved in the treatment of the patient's condition, the conception, and design of the study, final approval of the version to be submitted.

Hammad U. Qureshi: Primary surgeon involved in the treatment of the patient's condition. Responsible for contributing to the manuscript of the case report, final approval of the version to be submitted.

Abdullah AlHada: Assistant surgeon involved in the treatment of the patient's condition. Responsible for contributing to the manuscript of the case report.

Muhammed Sheikh: Assistant surgeon involved in the treatment of the patient's condition. Responsible for contributing to the manuscript of the case report.

## Registration of research studies

N.A.

## Guarantor

Ayman Khalil - a guarantor who accepts full responsibility for the work and the conduct of the study, has access to the data and controls the decision to publish.

## Provenance and peer review

Not commissioned, externally peer-reviewed.
